# Current Vaccine Trials in Glioblastoma: A Review

**DOI:** 10.1155/2014/796856

**Published:** 2014-04-03

**Authors:** Linda W. Xu, Kevin K. H. Chow, Michael Lim, Gordon Li

**Affiliations:** ^1^Department of Neurosurgery, Stanford University Medical Center, Stanford, CA 94304, USA; ^2^Department of Neurosurgery, Johns Hopkins University Medical Center, Baltimore, MD 21287, USA

## Abstract

Glioblastoma (GBM) is the most common primary brain tumor, and despite aggressive therapy with surgery, radiation, and chemotherapy, average survival remains at about 1.5 years. The highly infiltrative and invasive nature of GBM requires that alternative treatments for this disease be widespread and targeted to tumor cells. Immunotherapy in the form of tumor vaccines has the potential to meet this need. Vaccines against GBM hold the promise of triggering specific and systemic antitumor immune responses that may be the key to eradicating this unrelenting cancer. In this review, we will discuss past and present clinical trials of various GBM vaccines and their potential impact on the future care of GBM patients. There have been many promising phase I and phase II GBM vaccine studies that have led to ongoing and upcoming phase III trials. If the results of these randomized trials show a survival benefit, immunotherapy will become a standard part of the treatment of this devastating disease.

## 1. Introduction

Glioblastoma (GBM) is the most common primary brain tumor in humans, and despite recent advances in treatment, long-term survival remains low. The current standard of care includes surgical resection followed by concurrent radiation and temozolomide chemotherapy followed by adjuvant temozolomide [1, 2]. Median survival on this regimen has been reported to be approximately 1.5 years [1–3]. This therapy is nonspecific and almost invariably fails to prevent recurrence of disease. As the search for alternative and adjuvant treatment options continues, there is great interest in developing targeted immune-based therapies for GBM.

### 1.1. Cancer Immunotherapy

Cancer immunotherapy can be broadly defined as therapy that is based on the strategies employed by the body's immune system to eradicate malignant cells. Immunotherapy can be subcategorized as immunomodulator therapy, passive immunotherapy, or active immunotherapy. Immunomodulator therapy involves the administration of various interleukins, cytokines, and chemokines to activate or enhance the ability of endogenous immune effector cells to target and eradicate tumor cells. In melanoma, for instance, interleukin-2 (IL-2) and interferon (IFN)-*α* have become standard therapies as adjuvants to chemotherapy to enhance immune response in treating metastatic disease [4, 5].

Passive immunotherapy generally refers to the administration of monoclonal antibodies to target a specific antigen that is preferentially expressed on tumor cells. This allows for specific tumor killing with minimal toxicity to surrounding normal tissue. This type of targeted immunotherapy is already being widely used in humans in the form of Her2/neu antibodies for breast cancer [6–8]. Antibody therapy is considered passive since its efficacy is based on a direct effect of the administered antibody on tumor cells and does not primarily depend on activation of the body's immune system. Adoptive cellular therapy (ACT) is another type of immunotherapy that is also considered a passive strategy and involves the ex vivo culture of effector immune cells with subsequent transfer to the patient for a therapeutic response. ACT with various effector cells has been investigated in GBM patients and is reviewed elsewhere [9].

### 1.2. Cancer Vaccines

In contrast to antibodies, cancer vaccines are classified as active immunotherapy because they depend on activation of the patient's immune system to recognize and destroy the tumor. The advantage of this approach is the potential for eliciting a widespread and durable response. Vaccines directed towards cancer cells have been difficult to generate given the various mechanisms that are utilized by cancer cells to evade immune detection. A cancer vaccine directed towards metastatic prostate cancer has demonstrated modest success and has been approved by the FDA [10].

Factors to consider when designing or evaluating a cancer vaccine include the antigen(s) being targeted, the type of vaccine being tested, the specific adjuvant being used, and the method of vaccine delivery, as well as the efficacy of the vaccine given in combination with standard or other adjunct therapies (see Table 1). Central to the success of a vaccine is its ability to harness the potent antigen-presenting capabilities of dendritic cells (DCs). DCs, part of the innate immune system, incorporate antigens and subsequently present them to the cells of the adaptive immune system to initiate an immune response. DCs can be removed from the body and modified ex vivo to enhance specific antigen presentation or can be activated in vivo to the same end. In the former approach, everything from tumor cells, lysates, proteins, synthetic peptides, DNA, and RNA can be used to promote a DC-mediated antitumor response.

### 1.3. GBM Vaccines

Several studies of vaccines specific against GBM have been completed and more are underway (Tables 2 and 3). These vaccines utilize many of the general strategies listed above, with certain GBM-specific considerations. In particular, the identification of GBM-specific antigens has encouraged the development of vaccines specific to these antigens, such as the well characterized epidermal growth factor receptor variant 3 (EGFRvIII). Counter to this are strategies to target multiple antigens in GBM. This has generally been achieved through the use of lysates generated from resected tumors or synthetic peptide cocktails. Other strategies include specifically targeting cancer stem cell antigens or viral antigens of cytomegalovirus (CMV), which has been linked to GBM. In this review, we will analyze these topics in detail and offer our perspective on the future of GBM vaccines.

## 2. EGFRvIII Vaccines

Epidermal growth factor receptor (EGFR) plays a role in cellular processes such as migration, differentiation, and apoptosis [11]. Aberrant EGFR signaling is implicated in a variety of cancers, for which the receptor has served as a viable target. However, normal tissues also express EGFR and targeting all EGFRs can lead to unintended damage to normal tissue. One specific EGFR mutation, EGFRvIII, is found in 24–67% of GBMs but is not expressed in normal brain tissue [12, 13]. EGFRvIII mutations render the receptor constitutively active and contribute to uncontrolled cell proliferation and malignancy [14, 15]. As such, several groups have researched ways to target EGFRvIII in GBM. A peptide vaccine with synthetic EGFRvIII has been tested in phase I and phase II trials for newly diagnosed GBM.

In the VICTORI phase I trials for intracranial tumors, DCs loaded with EGFRvIII peptides were used for vaccination. Patients experienced minimal adverse reactions to the vaccine and over 60% became sensitized to the peptide on subsequent testing [16]. In 12 patients who received the vaccine, median progression-free survival was 10.2 months and median overall survival was 22.8 months from diagnosis [17]. A second study showed specific upregulation of T cells responsive to EGFRvIII and antibodies against the peptide after vaccination. Histologic analysis of recurrent tumors in patients who received the vaccine showed no residual expression of EGFRvIII [18], suggesting immunologic escape. In the phase II study, ACTIVATE, 19 patients with newly diagnosed EGFRvIII-expressing GBMs were treated with vaccine and granulocyte macrophage colony stimulating factor (GM-CSF), which has been shown to enhance immune responses to vaccination [19] (NCT00643097). In this study, progression-free survival was shown to be 12 months in comparison to 7.1 months in case control matched patients. In those patients who had subsequent serum samples that showed immune sensitization to EGFRvIII, overall survival was 47.7 months, compared with 22.8 months for those who did not show serum sensitization. Samples of recurring tumors showed no histological staining for EGFRvIII, suggesting complete eradication of EGFRvIII-containing cells [20]. During the ACTIVATE study, the results of the randomized trial demonstrated that temozolomide increased survival in newly diagnosed GBM patients. Therefore, temozolomide in addition to radiation became the new standard of care for newly diagnosed GBM patients [1]. ACT II, another phase II trial, was then started to compare the use of standard temozolomide doses of 200 mg/m^2^ for 5 days out of 4 weeks in conjunction with the EGFRvIII vaccine and GM-CSF versus temozolomide doses of 100 mg/m^2^ for 21 days out of 4 weeks in conjunction with the EGFRvIII vaccine and GM-CSF [21]. All immunized patients developed serum markers for sensitization to EGFRvIII. The patients who received the prolonged temozolomide dosing developed more severe lymphopenia; however, there was an even more robust serum immunity to EGFRvIII. The mechanism of the improved immune response may be related to a decrease in regulatory T cells and an increase in homeostatic cytokines, as was shown in patients undergoing adoptive transfer of T cells for metastatic melanoma and Epstein-Barr virus- (EBV-) associated nasopharyngeal carcinoma [22–24]. Overall, the patients showed improved survival compared with case controls with progression-free survival of 15.2 months and overall survival of 23.6 months. The larger ACT III trial with 65 patients showed a median progression-free survival of 12.3 months and overall survival of 24.6 months [19] (NCT00458601). This was initially done as a phase II/III randomized study; however, patients randomized to the nonvaccine arm dropped out.

The EGFRvIII peptide vaccine has now moved on to a phase III trial with ACT IV comparing vaccine plus GM-CSF and standard of care, against standard of care alone (NCT01480479). Patients are still being accrued to this study and the results are not complete. The phase II trial with relapsed GBM patients, ReACT, is also recruiting for administration of EGFRvIII peptide vaccine and GM-CSF in combination with bevacizumab (NCT01498328). There are two groups in this study. The first group is the bevacizumab naïve relapsed GBM patients. These patients are randomized to bevacizumab alone versus bevacizumab with the vaccine. The second group includes relapsed GBM patients who have already failed bevacizumab. These patients then receive the vaccine in addition to continuing with bevacizumab. Finally, at Stanford University, the EGFRvIII vaccination strategy is being tested in a phase I trial for children with diffuse intrinsic pontine gliomas where EGFRvIII expression was found in 50% of tumors studied [25] (NCT01058850). A summary of EGFRvIII vaccine trials can be found in Table 2.

The EGFRvIII vaccine trials demonstrate the safety and feasibility of administering a peptide vaccine directed to a single GBM-specific antigen. The recurrence of tumors lacking EGFRvIII expression suggests that the vaccine is effective in eradicating antigen-positive tumor cells but that immunologic escape portends tumor recurrence. GBM is characterized by extreme heterogeneity, which may render strategies directed at a single tumor antigen ineffective for long-term survival.

## 3. Tumor Lysate and Multipeptide-Specific Vaccines

Using whole tumor lysate as an antigen source has the advantage of providing a broad and patient-specific repertoire of potential immunologic targets. Briefly, tumor lysate vaccine is generated by culturing resected tumors ex vivo, isolating the surface proteins associated with major histocompatibility complexes (MHC), and combining them with autologous DCs. The first case study showing the feasibility of this approach was published in 2000, and a phase I trial of 12 patients was published in 2005 showing no major adverse events with the vaccine, as well as a small increase in survival and time to tumor progression without statistical significance [26, 27] (NCT00068510). In this trial, three vaccines were administered over several weeks and patients were followed for up to 5 years. Six patients who received the vaccine had peripheral blood samples that showed activated T cells against tumor cells, indicating a systemic response. Four out of 8 people who required reoperation had second tumor samples that showed T cell infiltration into the tumor [27]. Subsequent studies showed that those with low transforming growth factor-*β*2 (TGF-*β*2) expression had greater T cell infiltration, which also correlated with prolonged survival [27]. These findings suggest that low TGF-*β*2 levels may be a marker for increased susceptibility to tumor lysate DC vaccines.

Other studies have also shown that certain patients are more responsive to tumor lysate vaccines. Genetic analysis shows that gliomas can be divided into three categories of gene expression—proneural, proliferative, and mesenchymal [28]. Interestingly, although some studies have shown that the proneural subtype has a better survival outcome than the other subgroups [29, 30], the mesenchymal subgroup appears to respond best to tumor lysate DC vaccination with improved survival and improved T cell infiltration of the tumor [31] (NCT00068510). These lysate-pulsed DC vaccines have also been tested in pediatric patients with GBM. In a study of three patients, none experienced adverse outcomes from vaccine administration, and plans are underway for a phase I trial [32] (NCT01808820).

There are now several phase II trials and one phase III trial underway for evaluation of tumor lysate vaccines. The phase II trial from the same group that conducted the phase I study has been recruiting patients since 2010 and compares the efficacy of tumor lysate-pulsed DCs with two adjuvants—resiquimod, a topical agent to boost cytotoxic T cell response [33], and polyinosinic-polycytidylic acid stabilized by lysine and carboxymethylcellulose (poly-ICLC), an agent shown to promote T cell infiltration of gliomas [34] (NCT01204684). The phase III trial with the same group is beginning with two of three patients receiving vaccine in addition to standard radiation and temozolomide therapy and one of the three patients receiving placebo with the option of crossover to the vaccine group if disease progresses (NCT00045968). A phase II trial is also underway in Vienna that compares standard temozolomide and radiotherapy with temozolomide, radiotherapy, and a tumor lysate DC vaccine (NCT01213407). A third phase II trial looked at DCs loaded with tumor lysate antigens, matured by exposure to prostaglandin E2 (PGE2) and tumor necrosis factor-*α* (TNF-*α*), and injected directly into cervical lymph nodes (NCT00323115). Preliminary results from this study show that five patients had a systemic immune response to the vaccine with four of these five patients alive after 2 years, whereas none of the nonresponders survived to the 2-year mark [35]. Overall, tumor lysate DC vaccines have shown great promise over the last decade, serving as an adjuvant to traditional GBM treatments, especially for patients with certain subtypes of otherwise treatment-resistant GBMs.

While tumor lysates provide a large array of antigenic peptides, the immunogenicity of each antigen can vary widely depending on how efficiently they are bound to human leukocyte antigen (HLA) molecules and presented by DCs. This could result in inconsistent or suboptimal immune responses. This problem led a group in the United Kingdom to pursue peptide analysis to see what particular peptides in tumor cells were bound to HLA molecules and were not found in normal cells. In doing so, they identified 11 tumor-associated peptides (TUMAPs). An in vitro study showed T cell activation by presentation of these peptides [36]. DCs were then loaded with HLA complexes carrying these TUMAPs so they could present both these proteins and prime T cells for activation and proliferation and possibly create a robust immune response to tumor cells [37]. Preliminary results from a phase I trial show that eight of 11 patients had an immune response to the vaccine with no serious adverse effects to date (NCT01403285).

## 4. Tumor Stem Cell Vaccines

While tumor lysate can vaccinate against a variety of antigens that are expressed by GBM, it is possible that some antigens induce greater immunity than others and that greater efficacy can be achieved by targeting specific malignant cell types. Cancer stem cells are a subtype that has been identified within brain tumors. They can self-renew and can differentiate into a variety of other neural cells [38, 39]. These cells overexpress a variety of tumor-associated antigens, including HER2/neu, TRP-2, AIM-2, gp100, MAGE1, and IL13R*α*2 [40–45], and the presence of these cells is consistent with poor response to traditional GBM therapy [46–48]. In animal studies, DC vaccines using lysate from cancer stem cells rather than whole tumor lysate produced greater T cell responses [49]. Thus, ICT-107, a DC vaccine carrying the six synthetically created cancer stem cell-associated peptides, was created to target antigens specifically associated with aggressive cancer stem cells.

In a phase I trial, GBM patients were administered ICT-107 in conjunction with standard GBM therapy and results showed a nonstatistically significant increase in progression-free survival but no change in overall survival. Twenty-one patients were enrolled in the study and administered the vaccine three times over 6 weeks. Prevaccine genetic tests on the tumors showed that all patients expressed at least three of the antigens in the ICT-107 vaccine, and 75% expressed all six of the antigens within the vaccine. The median progression-free survival was 16.9 months and median survival was 38.4 months [50]. Interestingly, in those patients who required re-resection of tumor, the second tumor sample did show a decreased number of cancer stem cells, suggesting vaccine efficacy in targeting these aggressive cells within the tumor [40]. As these cells are poorly responsive to standard GBM therapy, it is possible that their reduction will improve susceptibility to current standard therapies. ICT-107 is currently in phase II trials with comparison against DCs without tumor antigens serving as a control in patients otherwise getting standard treatment with resection, radiation, and temozolomide (NCT01280552). A similar phase II trial using a tumor stem cell vaccine is ongoing in China (NCT01567202) [51].

## 5. CMV Vaccine

Several viruses have been associated with oncogenic potential, such as human papilloma virus with cervical cancer or EBV with lymphoma. Human CMV is the most common fetal infection, and in protein analysis, GBMs were found to produce CMV proteins [52]. In a model in which mice are prone to develop gliomas, infection with murine CMV greatly shortened survival and increased the percentage of GBMs instead of lower-grade tumors [53]. An initial clinical trial of a DC vaccine carrying RNA from human CMV was conducted. Thirteen patients were treated with the vaccine along with standard therapy. Median progression-free survival was 15.4 months and overall survival was 20.6 months [54]. The vaccine dubbed “PEP-CMV” is now going on to a multicenter trial (NCT01854099).

## 6. Enhanced DC Vaccines with Multiple Peptides

Different protocols for generating DCs for cancer vaccination may result in significant differences in the efficacy of generating T cell responses and ultimately in producing clinical responses in cancer patients [55]. The effectiveness of any given regimen depends on its ability to induce a robust type 1 immune response, which is critical for antitumor immunity [56]. Treatment of DCs with IL-4 has been shown to promote IL-12 production and promote type 1 immunity [57–60]. Two complementary clinical trials tested the use of IL-4 gene-transfected fibroblasts in combination with either autologous glioma cells alone or type 1 DCs loaded with autologous glioma lysate [61]. Twelve patients were enrolled in the two studies with 7/12 patients receiving one of the two vaccines. The remaining patients were withdrawn from the study due to tumor recurrence prior to completion of vaccine production, which took at least 7-8 weeks. As such, both trials were terminated early given major feasibility issues.

A subsequent clinical trial by the same group utilized new culture methods to further optimize DC function for optimal antitumor immunity [55, 62] (NCT00766753). Briefly, monocytes were cultured in GM-CSF and IL-4 to produce immature DCs, which were then polarized with IL-1*β*, TNF-*α*, IFN-*α*, IFN-*γ*, and poly-I:C, producing “*α*-type 1 polarized dendritic cells” (*α*DC1), which combine fully mature DC status with high migratory capability and enhanced IL-12 production. Twenty-two patients with recurrent malignant glioma (13 of whom had GBM) were treated with intranodal injections of *α*DC1 loaded with synthetic peptides for EphA2, IL13R*α*2, YKL-40, and gp100 HLA-A2 restricted epitopes in this phase I/II study. Poly-ICLC, which has been used in previous clinical trials to treat patients with GBM [63, 64], was administered intramuscularly as an adjuvant in accordance with data demonstrating its ability to boost postvaccination immune response and to promote T cell infiltration into the tumor [34, 65].

The treatment was well tolerated with minimal toxicity. The *α*DC1 from these patients produced a wide range of IL-12 levels, with higher levels correlating with longer time to progression. Nineteen patients completed the initial four vaccination course, and of those, 11 (58%) had immune reactivity to at least one of the four targeted antigens as demonstrated by IFN-*γ* ELISPOT assay or tetramer analysis on peripheral blood mononuclear cells (PBMCs). Upregulation of several type 1 cytokines and chemokines, including IFN-*α*, CXCL10, and IL-15, was shown in postvaccine analysis of patient PBMCs and serum samples. Two of 19 patients (11%) had objective clinical tumor regression (one partial response and one complete response) and 9 patients (41%; 4 GBM, 5 anaplastic glioma) were progression-free for at least 12 months.

## 7. In Vivo Recruitment of DCs

### 7.1. HSP-96 Vaccine

Generating DCs for cancer vaccination requires highly specialized cell culture techniques and ex vivo manipulation prior to obtaining the final product for injection. The antigen presenting capacity of a patient's DCs can be exploited for cancer vaccination without having to culture the cells ex vivo through the use of tumor-derived heat-shock proteins (HSPs) [66]. HSPs are a family of chaperone proteins whose physiologic function is in binding polypeptides and facilitating protein folding and transport. HSPs are upregulated by cellular stressors including heat (hence their name), hypoxia, infection, and malignant transformation. Additionally, HSPs are known to bind receptors expressed by DCs resulting in the delivery of a broad array of peptides to the body's most efficient antigen presenters, which can then go on to prime CD4 and CD8 T cell responses.

HSP-96 from GBM has been investigated by Crane et al. [67] for use as a cancer vaccine. Twelve patients with recurrent GBM were treated in this phase I study with intradermal injections of autologous tumor-derived HSP peptide complex (HSPPC), a complex consisting of HSP-96 and a broad array of tumor-associated antigenic peptides. The vaccine was prepared by Agenus Incorporated (New York, NY, USA) through a proprietary procedure. Patients received at least four 25 *μ*g doses of HSPPC-96 every 1–3 weeks, with subsequent immunomonitoring, imaging, and postvaccine biopsy. Eleven of 12 patients were reported to have specific peripheral immune responses as determined by in vitro restimulation of bulk peripheral blood lymphocytes with vaccine and testing for cytokine expression and proliferation. Seven of 12 patients had a postvaccine tumor resected, and of those, all had increased immune cell infiltrates. The one patient who did not have a detectable response to the vaccine had a shorter survival time than the 11 responders and was noted to have a higher tumor burden and circulating levels of T regulatory cells. A phase II trial of this treatment has completed accrual and is in the follow-up phase (NCT00905060, NCT00293423). In addition, there is a phase II trial underway to test HSPPC-96 with bevacizumab versus bevacizumab alone in patients with recurrent GBM (NCT01814813).

### 7.2. Irradiated Tumor Cells

An alternative to using DCs loaded ex vivo with antigen-rich tumor lysates is the direct injection of irradiated tumor cells. Irradiation ensures that the tumor cells can no longer grow and are used for stimulating the patient's DCs in vivo. This method has been described previously for other tumors [68]. The irradiated tumor cells are injected in conjunction with K562/GM-CSF cells that oversecrete GM-CSF, which stimulates the recruitment of DCs to the site of injection. This has been tested in a number of cancers and is now entering a phase I trial in patients with GBM (NCT00694330).

## 8. Discussion

In this review we have discussed the different vaccination strategies for the treatment of GBM and reviewed the numerous trials that are completed and ongoing. While there have been some moderate increases in survival in clinical trials with the various tumor vaccine strategies and appropriate immunologic responses, all of the results to date have been compared to historical controls. It is unclear which of these strategies will lead to the most durable and potent increase in overall survival. Each trial has its own shortcomings. For example, the HSP vaccine and DC vaccine trials require tumor tissue from resection and a significant amount of tissue processing. The ICT107 trial requires a specific HLA type, and the EGFRvIII vaccine requires the EGFRvIII mutation. We look forward to the randomized trials that are underway which will more definitively show whether vaccination strategies are a viable method for the treatment of GBM. ACT IV, the randomized trial studying the EGFRvIII vaccine in newly diagnosed GBM, is the closest to accrual and if there is a positive result, it may become the first FDA approved immunotherapy strategy for brain tumors. The other phase III GBM immunotherapy trial is the DCVax (dendritic cell vaccine) trial. Many of the phase II studies have been promising and more phase III trials are currently being planned.

Although we are still awaiting the phase III trial results, we have learned much from the phase I and II trials. All of the GBM cancer vaccine trials have been shown to be safe. Theoretically, one could be concerned that the immune reaction against a tumor in the brain could lead to an inflammatory or autoimmune reaction in the central nervous system. This has not been an issue in these trials. In addition, the brain has traditionally been thought of as an immune privileged organ, meaning that the brain is protected from immune cells. If this is the case, immunotherapy for GBM should not be effective. However in these trials, not only have there been specific immune responses detected in the peripheral blood, but there has also been evidence of immune cells reaching the tumor. For example, in the DCVax trials, there was evidence of T cells in re-resected tumors. Many of the trials have correlated peripheral immune response to survival, suggesting that the immune response is involved in tumor killing. Finally, in the EGFRvIII vaccine trials, patients who underwent re-resection no longer expressed EGFRvIII, demonstrating that the vaccination strategy is effective. However, targeting multiple antigens may be necessary to avoid immunologic escape. Another potential issue for immunotherapy is that chemotherapy, steroids, and bevacizumab all suppress the immune system. Although more data are needed, preliminary results suggest that these agents cannot completely abrogate immunotherapy. In the ACT II trial, patients who received the higher dose of temozolomide experienced expected lymphopenia. However, these patients had increased EGFRvIII antibodies, potentially because of a decrease in T regulatory cells. In the ReACT trial, patients who were given bevacizumab still mounted an EGFRvIII-specific immune response.

Choosing the correct antigen and vaccination strategy is extremely important. None of the trials described in this review have come close to curing GBM, indicating that further refinements in target selection and vaccination strategy are necessary. In addition, the GBM microenvironment has multiple mechanisms for immune suppression that may limit the success of current vaccines. Concurrent research is being conducted to investigate adjuvant agents that may enhance response to immunotherapy. If one could choose an appropriate target and vaccination strategy and combine an immune modulator that could either suppress the anti-immune defense of the tumor or increase the body's ability to mount an immune response against the tumor, immunotherapy perhaps could lead to even more durable responses in GBM patients. There are a number of trials currently being planned to test these immunomodulators.

## Figures and Tables

**Table 1:** Vaccine strategies.

Products	Comments	
Whole tumor cell	Broad range of antigens, known and unknown	
	*Autologous *	Patient-specific, customized, and of high cost of production
	*Allogeneic *	Based on one or more tumor cell lines, “off the shelf,” and easier to produce
	*Genemodified *	Increase antitumor immunity, cytokine expression (IL-2, GM-CSF), and costimulatory molecules (B7-1)
Dendritic cell	Most potent antigen presenting cell, highly specialized culture techniques	
	*Tumor pulsed *	Broad array of antigens
	*Peptide/protein pulsed *	Single or combination of antigens targeted, highly specific
	*Genemodified *	Expression of cytokines or costimulatory molecules to enhance immunogenicity
Protein	Single or combination, potential for autoimmunity	
Peptide	Minimize autoimmunity associated with whole protein and are easy to produce, cost-effective, and able to enhance immunogenicity and to quantify peptide specific T cell response with tetramer	
Heat-shock proteins	Purified from tumor cells, immune response to peptides carried by the HSPs	
Other	Viral and bacterial vectors, plasmid DNA	

Adjuvants		
TLR agonists	IFA (incomplete Freund's adjuvant), BCG, LPS (lipopolysaccharide), RNA, CpG DNA motifs, and MPL (monophosphoryl lipid A)	
Cytokines	IL-2, GM-CSF	
Costimulatory molecules	B7-1, B7-2, and CD40	

Delivery		
Intradermal	Easy to administer and requires migration of DCs to draining lymph node or scavenging of antigens by endogenous DCs	
Intranodal	Theoretical advantage of bypassing need for lymph node migration, possible destruction of LN architecture, and questionable benefit	
Intratumoral	Enhance immunogenicity of tumor and may not be feasible for brain tumors	

Combinatorial strategies		
STAT3 inhibition	Reverse tumor induced STAT3 mediated immunosuppression	
PD-1 blockade	Enhance CD8 T cell function, effective in non-small-cell lung CA, melanoma, and renal cell CA	
Chemotherapy and radiation	Potential for upregulation of tumor antigens and MHC and decreased tumor burden	

**Table 2:** EGFRvIII vaccine trials.

Trial name	Phase	*N*	Experimental design	PFS (mo)	OS (mo)	References
VICTORI	I	12	Vaccine administered 60% sensitized	10.2	22.8	[16, 17]

ACTIVATE	II	19	Vaccine + GM-CSF versus case matched controls	12	47.7 in sensitized pts; 22.8 in nonsensitized pts	NCT00643097 [20]

ACT II	II	22	Vaccine + high dose short course TMZ versus low dose prolonged course TMZ. Improved immune sensitization in patients with prolonged TMZ treatment	15.2	23.6	[21]

ACT III	II	65	Vaccine + GM-CSF + TMZ versus TMZ	12.3	24.6	NCT00458601 [19]

ACT IV	III		Vaccine + GM-CSF + TMZ versus TMZ and placebo alone	Ongoing	Ongoing	NCT01480479

ReACT	II		Relapsed GBM, vaccine + GM-CSF + bevacizumab versus bevacizumab	Ongoing	Ongoing	NCT01498328

Pediatric pontine glioma pilot study	I		Children with DIPG vaccine + GM-CSF after radiation	Ongoing	Ongoing	NCT01058850

*N*: number of patients; PFS: progression-free survival; OS: overall survival.

**Table 3:** GBM vaccine trials.

Vaccine type	Phase	*N*	Experimental design	PFS (mo)	OS (mo)	References
Tumor lysate vaccine	I	12	Autologous DC loaded with tumor lysate	15.5	23.4	NCT00068510, [27]

Tumor lysate vaccine	II		Resiquimod, poly-ICLC	Ongoing	Ongoing	NCT01204684

DCVax-Brain	III		2/3 vaccine, 1/3 placebo with option of crossover at disease progression	Ongoing	Ongoing	NCT00045968

Tumor lysate vaccine	II		Vaccine + standard therapy versus standard therapy alone	Ongoing	Ongoing	NCT01213407

Tumor lysate vaccine	II	10	DCs treated with PGE2 and TNF-*α*, cervical lymph node injection	9.5	28	NCT00323115, [35]

IMA950 multipeptide vaccine	I		11 tumor associated peptides (TUMAPs) + GM-CSF, cyclophosphamide, imiquimod	Ongoing	Ongoing	NCT01403285

Cancer stem cell vaccine, ICT-107	I	21	Six synthetic peptides associated with CSCs loaded onto autologous DCs	16.9	38.4	[50]

Cancer stem cell vaccine, ICT-107	II		Autologous DCs pulsed with immunogenic peptides from tumor antigens versus placebo	Ongoing	Ongoing	NCT01280552

Cancer stem cell vaccine	II		Autologous DCs loaded with stem cell-like antigens from irradiated GBM versus placebo	Ongoing	Ongoing	NCT01567202

CMV vaccine (Pep-CMV)	I		Intradermal Pep-CMV following chemoradiation	Ongoing	Ongoing	NCT01854099

Alpha type I DC peptide vaccine	I/II	22	Four peptides loaded onto alpha type I DCs + poly-ICLC, included GBM and anaplastic glioma	4 in GBM 13 in anaplastic glioma		NCT00766753, [61, 62]

HSPPC-96	I	12	Autologous tumor derived HSPPC-96 administered intradermally		47 weeks in immune responders 16 weeks in nonresponder	[67]

HSPPC-96	II		Autologous tumor derived HSPPC-96 administered intradermally	Ongoing	Ongoing	NCT00905060, NCT00293423

HSPPC-96	II		Vaccine + bevacizumab versus bevacizumab alone	Ongoing	Ongoing	NCT01814813

Irradiated glioma cells with GM-K562	I		Admixture of lethally irradiate glioma cells with GM-CSF producing K562 injected intradermally	Ongoing	Ongoing	NCT00694330
